# Symptomatic intraocular pressure dysregulation during clopidogrel exposure after patent foramen ovale closure in a patient with ocular hypertension and angle crowding: a Case Report

**DOI:** 10.3389/fmed.2026.1836893

**Published:** 2026-07-16

**Authors:** Faxue Li, Rongbo Yu, Xuan Guo, Wenyi Ji, Qiang Wu

**Affiliations:** Department of Cardiology, Lanzhou University Second Hospital, Lanzhou, Gansu, China

**Keywords:** angle crowding, case report, clopidogrel, diurnal fluctuation, intraocular pressure, ocular hypertension, patent foramen ovale closure, pharmacovigilance

## Abstract

Clopidogrel is widely used after structural heart interventions, but symptomatic intraocular pressure (IOP) abnormalities without clinically evident hemorrhage are rarely reported. We describe a 52-year-old woman who developed recurrent ocular discomfort, eye pain, and blurred vision after percutaneous patent foramen ovale closure followed by clopidogrel-containing antiplatelet therapy. Baseline pre-exposure assessment showed ocular hypertension with angle crowding features but no definite glaucomatous structural or functional damage. Symptoms improved after clopidogrel withdrawal and recurred after re-administration. During recurrent symptomatic exposure, 24-h IOP monitoring showed marked peak elevation and diurnal fluctuation in both eyes. Baseline pre-exposure IOP measurements were available, but baseline 24-h IOP monitoring was not performed; therefore, worsening relative to the patient's baseline diurnal pattern could not be confirmed. Formal gonioscopy and symptomatic-phase angle reassessment were unavailable, and intermittent angle closure could not be excluded. This case represents a dechallenge–rechallenge-supported temporal association and hypothesis-generating pharmacovigilance signal rather than definitive evidence of a clopidogrel-induced effect.

## 1 Introduction

Clopidogrel is a commonly used P2Y12 receptor inhibitor for antiplatelet therapy in coronary artery disease, ischemic stroke, and after structural heart interventions (1, 2). Reported ocular adverse events associated with clopidogrel have pre-dominantly been hemorrhagic, including vitreous or choroidal hemorrhage and related secondary intraocular pressure (IOP) elevation, whereas reports of non-hemorrhagic ocular adverse events directly linked to clopidogrel are sparse and, to our knowledge, have not clearly described symptomatic 24-h IOP dysregulation with recurrence after rechallenge (3–5). Because elevated peak IOP and increased diurnal fluctuation are clinically relevant risk factors for glaucoma onset and progression (6–8), recognition of medications that may aggravate IOP abnormalities is important in susceptible patients.

Here, we report a 52-year-old woman with ocular hypertension and angle crowding features who developed recurrent ocular symptoms during clopidogrel exposure after percutaneous patent foramen ovale closure. Twenty-four-hour IOP monitoring documented marked peak elevation and diurnal fluctuation during recurrent symptomatic exposure, without clinically evident retinal or optic disc hemorrhage on available imaging. Because baseline 24-h IOP monitoring, formal gonioscopy, and symptomatic-phase angle reassessment were unavailable, this report is presented as a hypothesis-generating temporal association rather than evidence of a definitive drug-induced effect.

## 2 Case presentation

### 2.1 Patient information

The patient was a 52-year-old Asian woman who presented on October 12, 2024, with intermittent chest tightness for more than 10 years. She was subsequently diagnosed with patent foramen ovale, and percutaneous patent foramen ovale closure was planned. She had no previous definite diagnosis of glaucoma, no history of ocular trauma or ophthalmic surgery, and no definite family history of glaucoma. She also denied recent use of corticosteroids, mydriatic agents, anticholinergic drugs, or other medications known to induce intraocular pressure elevation.

### 2.2 Pre-operative ophthalmic assessment

During hospitalization on October 14, 2024, the patient underwent ophthalmic consultation because of left eyelid discomfort and ocular dryness. Examination showed an uncorrected visual acuity of 0.22 logMAR in the right eye and 0.10 logMAR in the left eye, with best-corrected visual acuity of approximately 0.10 logMAR in both eyes. Both corneas were clear. The central anterior chamber depth was acceptable, whereas the peripheral anterior chamber was relatively shallow. The aqueous humor was clear, the pupils were equal and round with preserved light reflexes, and lens transparency was mildly reduced. Fundus examination showed a cup-to-disc ratio of approximately 0.4 under small pupils, clear optic disc margins, a preserved foveal reflex, and an attached retina within the visible range.

**Figure 1:**
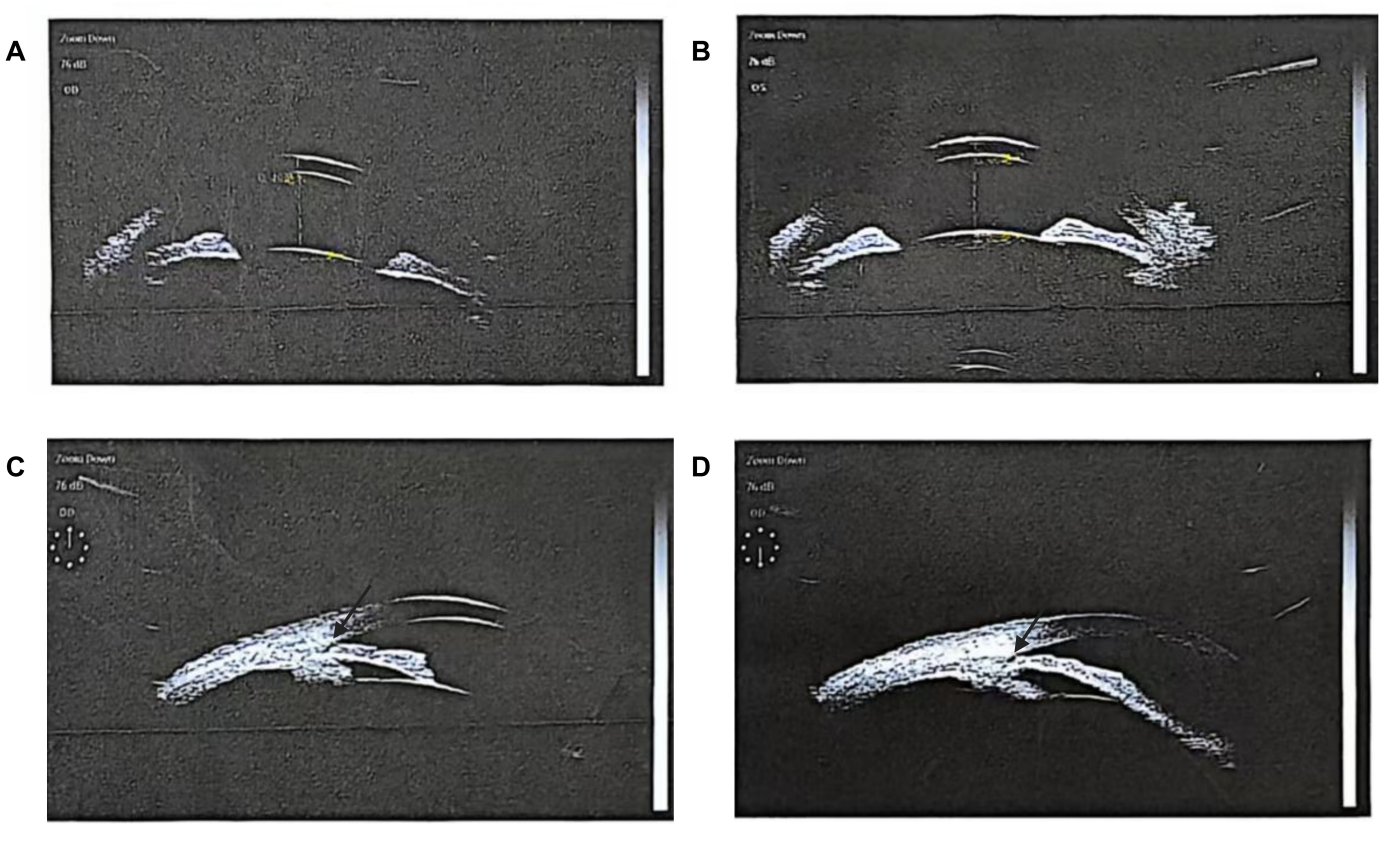
Baseline ultrasound biomicroscopy findings before clopidogrel exposure. Ultrasound biomicroscopy was performed on October 14, 2024, before clopidogrel exposure. **(A)** Overview of the right-eye ultrasound biomicroscopy examination. **(B)** Overview of the left-eye ultrasound biomicroscopy examination, showing open anterior chamber angles in the available quadrants. **(C, D)** Representative right-eye images at the 12 o'clock and 6 o'clock positions, respectively, showing angle crowding. These findings suggested quadrant-dependent angle crowding, particularly in the right eye. Formal gonioscopy with Shaffer grading and peripheral anterior synechiae assessment was not performed.

**Figure 2:**
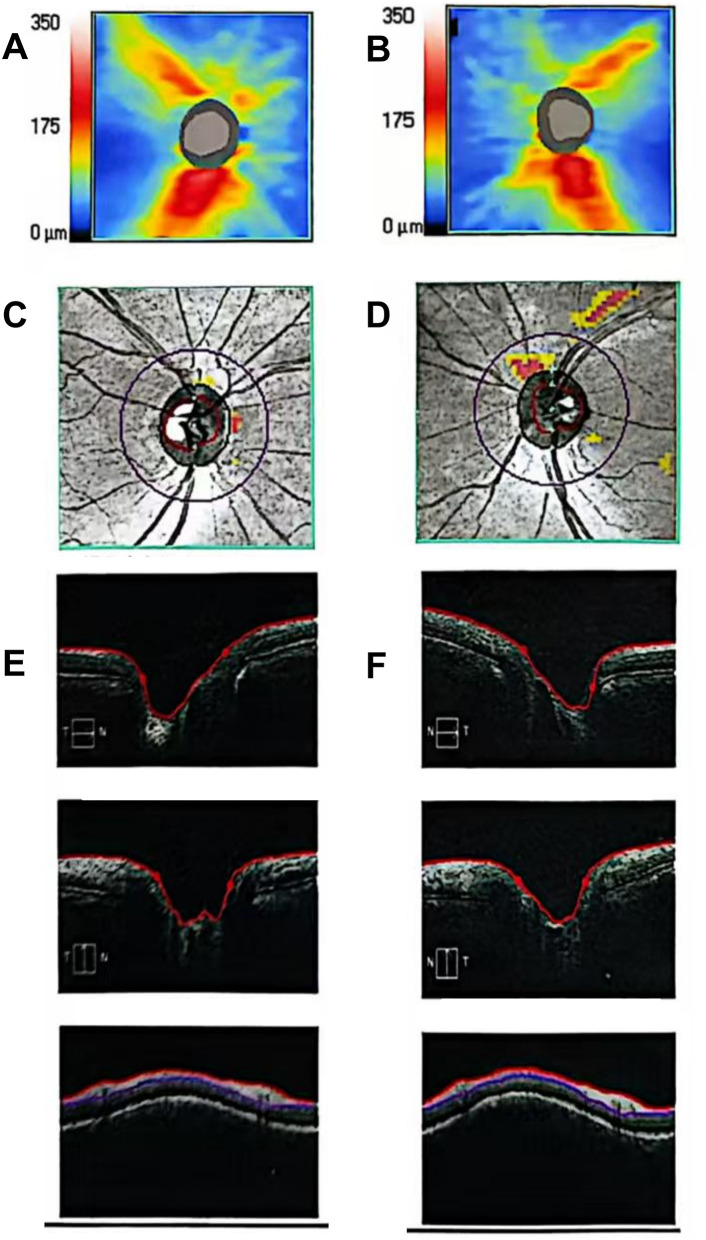
Baseline OCT RNFL/ONH assessment before clopidogrel exposure. OCT RNFL/ONH analysis was performed on October 14, 2024, before clopidogrel exposure. **(A, B)** RNFL thickness maps of the right eye (OD) and left eye (OS), respectively. **(C, D)** Optic disc-centered RNFL deviation maps of OD and OS, respectively. **(E, F)** Representative OCT B-scan images of OD and OS, respectively, including optic disc horizontal/vertical scans and peripapillary RNFL circular scans. No definite glaucomatous structural damage was observed in either eye, supporting classification as ocular hypertension with angle crowding rather than established glaucomatous optic neuropathy.

Goldmann applanation tonometry documented IOP values of approximately 23.0 mmHg in the right eye and 23.2 mmHg in the left eye. Corvis ST examination showed biomechanically corrected IOP values of approximately 17.0 mmHg in the right eye and 20.6 mmHg in the left eye, with central corneal thickness values of 531 and 524 μm, respectively (Supplementary Figure S2). Ultrasound biomicroscopy showed anatomical features suggestive of angle crowding, particularly in the right eye, whereas the left eye showed relatively open angles in the available images or quadrants (Figure 1). However, formal gonioscopy with Shaffer grading and peripheral anterior synechiae assessment was not performed. Optical coherence tomography retinal nerve fiber layer and optic nerve head analysis showed no definite glaucomatous structural damage in either eye (Figure 2), and Humphrey 24-2 visual field testing showed no definite glaucomatous functional defect (Supplementary Figure S1). Baseline 24-h IOP monitoring was not performed. Because the ophthalmic assessment was performed as a routine pre-operative consultation rather than a prospective IOP-monitoring protocol, baseline 24-h IOP monitoring was not clinically obtained at that time, and the specific reason for not performing it was not documented in the available clinical record. Taken together, these findings supported baseline classification as ocular hypertension with angle crowding features, without definite glaucomatous structural or functional damage, rather than established glaucomatous optic neuropathy.

### 2.3 Clinical course and timeline

On October 16, 2024, the patient underwent percutaneous patent foramen ovale closure and received post-operative therapy with aspirin 100 mg/day, atorvastatin 20 mg/day, and clopidogrel 75 mg/day. Approximately 2 weeks after surgery, she gradually developed recurrent ocular discomfort, eye pain, and blurred vision with fluctuating intensity. She therefore visited the ophthalmology outpatient clinic on December 4, 2024, and December 13, 2024. Based on the temporal relationship between symptom onset and medication exposure, discontinuation of clopidogrel was clinically recommended. Within approximately 2 weeks after clopidogrel withdrawal, ocular discomfort and eye pain gradually improved.

**Figure 3:**
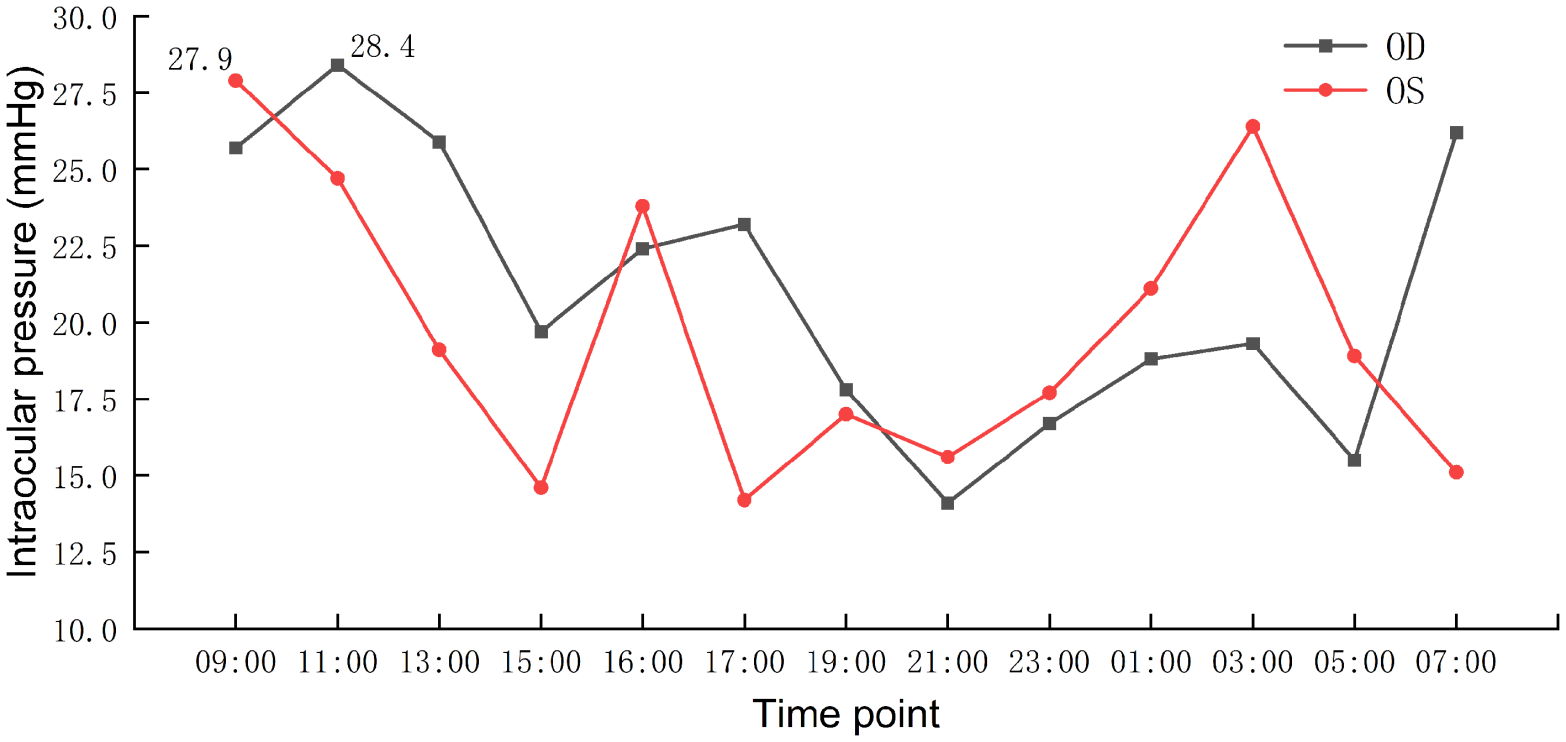
Twenty-four-hour intraocular pressure fluctuation during recurrent symptomatic clopidogrel exposure. Twenty-four-hour IOP monitoring showed peak IOP values of 28.4 mmHg OD and 27.9 mmHg OS, with peak-to-trough fluctuations of 14.3 and 13.7 mmHg, respectively. Baseline 24-h IOP monitoring before clopidogrel exposure was unavailable; therefore, worsening relative to the patient's baseline diurnal fluctuation pattern could not be confirmed.

On December 31, 2024, the patient resumed clopidogrel, and similar ocular symptoms recurred approximately 15 days later. On January 24, 2025, during recurrent symptomatic clopidogrel exposure, 24-h IOP monitoring at the ophthalmic center showed a maximum of 28.4 mmHg, a minimum of 14.1 mmHg, and a fluctuation of 14.3 mmHg in the right eye, and a maximum of 27.9 mmHg, a minimum of 14.2 mmHg, and a fluctuation of 13.7 mmHg in the left eye. Measurements obtained at different time points showed marked peak-to-trough changes in both eyes, documenting pronounced peak intraocular pressure elevation and marked diurnal fluctuation during the monitored symptomatic period (Figure 3); the detailed time-point-specific results are provided in Supplementary Figure S4. During 24-h intraocular pressure monitoring, IOP was measured by Goldmann applanation tonometry at each scheduled time point. Baseline pre-exposure IOP measurements were available; however, baseline 24-h IOP monitoring was not performed, so worsening relative to the patient's baseline diurnal fluctuation pattern could not be confirmed. Given concomitant aspirin therapy, the recent cardiovascular intervention, and the post-operative context, the specific contribution of clopidogrel could not be isolated. The medication exposure, symptom course, dechallenge–rechallenge pattern, and IOP-related findings are summarized in Table 1.

**Table 1:** Timeline of clopidogrel exposure, ocular symptoms, dechallenge–rechallenge course, and intraocular pressure-related findings.

Time point	Clopidogrel exposure and concomitant medications	Ocular symptoms	IOP-related and ophthalmic findings
October 14, 2024	Clopidogrel had not yet been started.	Left eyelid discomfort and ocular dryness were noted during pre-operative ophthalmic consultation.	Afternoon Goldmann applanation tonometry showed IOP of approximately 23.0 mmHg OD and 23.2 mmHg OS. Corvis ST showed biomechanically corrected IOP of approximately 17.0 mmHg OD and 20.6 mmHg OS, with central corneal thickness of 531 μm OD and 524 μm OS. Baseline findings were most consistent with ocular hypertension with angle crowding features, without definite glaucomatous structural or functional damage.
October 16, 2024	Percutaneous PFO closure was performed. Post-operative therapy included aspirin 100 mg/day, clopidogrel 75 mg/day, and atorvastatin 20 mg/day.	No immediate post-operative ocular worsening was documented.	This time point marked the start of clopidogrel-containing antiplatelet therapy.
Approximately 2 weeks after surgery	Clopidogrel-containing therapy continued.	Recurrent ocular discomfort, eye pain, and blurred vision gradually developed, with fluctuating intensity.	Symptoms developed after initiation of clopidogrel-containing therapy.
December 4 and December 13, 2024	Clopidogrel was still being used before withdrawal was recommended.	The patient visited the ophthalmology outpatient clinic multiple times because of recurrent ocular symptoms.	Based on the temporal relationship between medication exposure and symptoms, discontinuation of clopidogrel was clinically recommended.
After clopidogrel withdrawal	Clopidogrel was discontinued. Aspirin could not be discontinued because of the perioperative antiplatelet requirement.	Ocular discomfort and eye pain gradually improved within approximately 2 weeks.	Symptom improvement after withdrawal supported a temporal dechallenge pattern, although additive or synergistic effects of dual antiplatelet therapy could not be excluded.
December 31, 2024	Clopidogrel was re-administered.	No immediate symptom recurrence was documented on the day of re-exposure.	This time point marked the rechallenge.
Approximately 15 days after re-exposure	Clopidogrel exposure continued.	Similar ocular symptoms recurred.	Symptom recurrence after re-administration supported a temporal rechallenge pattern, but did not establish causality.
January 24, 2025	During recurrent symptomatic clopidogrel exposure.	The patient was symptomatic.	Twenty-four-hour IOP monitoring by Goldmann applanation tonometry showed maximum/minimum/fluctuation of 28.4/14.1/14.3 mmHg OD and 27.9/14.2/13.7 mmHg OS. Detailed time-point-specific measurements are provided in Supplementary Figure S4.
During follow-up	Atorvastatin was discontinued for 1 week without obvious symptom improvement. After clopidogrel was discontinued again, antiplatelet therapy was later adjusted to ticagrelor 90 mg twice daily plus aspirin 100 mg once daily.	Ocular symptoms gradually improved after clopidogrel discontinuation.	No cardiovascular adverse events were reported through February 20, 2026.

IOP, intraocular pressure; OD, right eye; OS, left eye; PFO, patent foramen ovale.

Baseline 24-h IOP monitoring before clopidogrel exposure was unavailable; therefore, worsening relative to the patient's baseline diurnal fluctuation pattern could not be confirmed. Formal gonioscopy and symptomatic-phase angle reassessment were not performed, so intermittent angle closure or other dynamic angle-related mechanisms could not be excluded. Given the recent PFO closure, concomitant aspirin therapy, and post-operative context, the specific contribution of clopidogrel could not be isolated.

## 3 Diagnostic assessment

### 3.1 Key examination findings

Baseline structural and functional testing showed no definite glaucomatous structural damage or functional defect on optical coherence tomography retinal nerve fiber layer/optic nerve head analysis and Humphrey 24-2 visual field testing (Figure 2, Supplementary Figure S1). Corvis ST provided baseline IOP and corneal biomechanical parameters (Supplementary Figure S2). Baseline ultrasound biomicroscopy demonstrated anatomical features suggestive of quadrant-dependent angle crowding in the right eye, whereas the left anterior chamber angles were open in the available quadrants (Figure 1). Formal gonioscopy with Shaffer grading and peripheral anterior synechiae assessment was not performed, and angle configuration was not reassessed during the symptomatic phase. The available symptomatic-period records did not document formal gonioscopy or repeat ultrasound biomicroscopy, and the specific reason for not obtaining these examinations during symptomatic visits could not be determined retrospectively. Therefore, intermittent angle closure or other dynamic angle-related mechanisms could not be confidently excluded. Ultra-widefield fundus photography showed no clinically evident retinal or optic disc hemorrhage on available imaging (Supplementary Figure S3), although occult hemorrhagic or subtle structural changes could not be fully excluded because B-scan ultrasonography, symptomatic-phase ultrasound biomicroscopy, and macular optical coherence tomography were not performed. During the recurrent symptomatic period, 24-h IOP monitoring demonstrated marked peak IOP elevation and diurnal fluctuation in both eyes (Figure 3, Supplementary Figure S4). Baseline pre-exposure IOP measurements were available, but baseline 24-h IOP monitoring was not performed; therefore, worsening relative to the patient's baseline diurnal fluctuation pattern could not be confirmed.

### 3.2 Diagnostic interpretation

Baseline findings were most consistent with ocular hypertension with angle crowding features rather than established glaucomatous optic neuropathy. On this background, the patient developed recurrent ocular symptoms approximately 2 weeks after initiation of clopidogrel-containing antiplatelet therapy. Symptoms improved after withdrawal and recurred after re-exposure, while 24-h IOP monitoring during recurrent symptomatic exposure objectively documented elevated peak IOP and marked diurnal fluctuation in both eyes. However, because baseline 24-h IOP monitoring was unavailable, worsening relative to the patient's baseline diurnal IOP pattern could not be confirmed. In addition, because formal gonioscopy and symptomatic-phase angle reassessment were not performed, intermittent angle closure or other dynamic angle-related mechanisms could not be excluded. Taken together, these findings support a dechallenge–rechallenge-supported temporal association in a susceptible patient, but do not establish a definitive clopidogrel-induced effect.

### 3.3 Temporal and pharmacovigilance assessment

The Naranjo Adverse Drug Reaction Probability Scale yielded a total score of 6 according to this structured assessment tool (9). The main positive items were the temporal sequence after clopidogrel exposure (+2), symptom improvement after withdrawal (+1), recurrence after re-administration (+2), and objective confirmation by 24-h intraocular pressure monitoring during the recurrent symptomatic period (+1). However, this structured assessment was interpreted cautiously because Naranjo-based causality assessment has limited specificity in isolated case reports, particularly when pre-existing ocular hypertension, angle crowding, recent cardiovascular intervention, post-operative physiological stress, and concomitant antiplatelet therapy may independently contribute to the observed findings. Alternative causes were therefore not considered excluded, and the Naranjo score was used only as a pharmacovigilance aid to describe the dechallenge–rechallenge-supported temporal pattern. Overall, this assessment supports a possible temporal association at the case-report level, but does not establish definitive causality.

### 3.4 Differential diagnosis

First, the clinical course should be differentiated from the natural fluctuation of ocular hypertension with angle crowding features. Because the patient already had elevated IOP and angle crowding before surgery, natural fluctuation remains an important alternative explanation (6, 7). In addition, baseline 24-h IOP monitoring was unavailable, so worsening relative to the patient's baseline diurnal fluctuation pattern could not be confirmed.

Second, typical acute angle-closure crisis was not documented (10, 11). Although the patient had quadrant-dependent angle crowding in the right eye and a relatively shallow peripheral anterior chamber, the left anterior chamber angles were open in the available quadrants, and there were no typical signs of acute angle closure such as corneal edema or a fixed mid-dilated pupil. However, formal gonioscopy with Shaffer grading and peripheral anterior synechiae assessment was not performed, and the angle configuration was not reassessed during the symptomatic phase. Therefore, intermittent angle closure or other dynamic angle-related mechanisms could not be excluded.

Third, hemorrhagic secondary ocular hypertension should be considered. Previous reports have mainly linked clopidogrel-associated IOP elevation to hemorrhagic complications (4, 5). In the present case, no clinically evident intraocular hemorrhage was identified on available examinations, although occult hemorrhagic mechanisms could not be definitively excluded because B-scan ultrasonography, symptomatic-phase ultrasound biomicroscopy, and macular optical coherence tomography were not performed.

Other potential confounders were considered with different levels of plausibility. Underlying ocular hypertension with angle crowding and ongoing aspirin co-administration were the most difficult to separate from clopidogrel exposure because they persisted throughout much of the clinical course. Post-operative physiological stress and natural IOP variability remained plausible but temporally nonspecific explanations. Atorvastatin was temporarily discontinued without obvious symptom improvement, arguing against a dominant role of atorvastatin but not fully excluding its contribution. Overall, these alternatives require cautious interpretation of the case as a hypothesis-generating temporal association rather than proof of a clopidogrel-specific effect.

## 4 Treatment, follow-up, and outcomes

The patient initially received aspirin, atorvastatin, and clopidogrel after the procedure. Because recurrent ocular symptoms developed after surgery and were temporally related to medication exposure, discontinuation of clopidogrel was recommended. Aspirin could not be discontinued because of the perioperative antiplatelet requirement; therefore, the potential contribution of aspirin co-administration or dual antiplatelet therapy could not be excluded. Ocular discomfort and eye pain gradually improved after clopidogrel withdrawal and recurred after re-exposure, supporting a temporal dechallenge–rechallenge pattern but not establishing causality.

During follow-up, atorvastatin was discontinued for 1 week without obvious symptom improvement, arguing against a dominant role of atorvastatin but not fully excluding its contribution. After clopidogrel was discontinued again, ocular symptoms gradually improved. At follow-up through February 20, 2026, clopidogrel remained discontinued, and antiplatelet therapy had been adjusted to ticagrelor (90 mg twice daily) plus aspirin (100 mg once daily), with no cardiovascular adverse events reported.

## 5 Discussion

This case describes recurrent ocular symptoms and objectively documented marked 24-h IOP fluctuation during clopidogrel exposure after patent foramen ovale closure in a patient with pre-existing ocular hypertension and angle crowding features. As summarized in Table 1, symptoms improved after clopidogrel withdrawal and recurred after clopidogrel re-administration, while 24-h IOP monitoring during the recurrent symptomatic exposure period documented peak IOP values of 28.4 mmHg and 27.9 mmHg in the right and left eyes, respectively. Greater IOP variability has been associated with faster glaucomatous progression (12, 13). However, because baseline 24-h IOP monitoring was unavailable, the documented fluctuation during recurrent symptomatic exposure could not be confirmed to represent worsening relative to the patient's baseline diurnal IOP pattern. In addition, because formal gonioscopy and symptomatic-phase angle reassessment were not performed, intermittent angle closure or other dynamic angle-related mechanisms could not be excluded. Therefore, this case should be interpreted as a dechallenge–rechallenge-supported temporal association and hypothesis-generating pharmacovigilance signal rather than evidence of a definitive clopidogrel-induced effect.

Previous reports of clopidogrel-associated ocular adverse events have mainly focused on hemorrhagic complications (3–5). In the present case, no clinically evident retinal or optic disc hemorrhage was identified on available ultra-widefield fundus photography. Nevertheless, occult hemorrhagic or subtle structural changes could not be fully excluded because B-scan ultrasonography, symptomatic-phase ultrasound biomicroscopy, and macular OCT were unavailable. Several alternative explanations also remain plausible, including natural IOP variability on a background of ocular hypertension and angle crowding, recent cardiovascular intervention, post-operative physiological stress, aspirin co-administration, dual antiplatelet therapy, and atorvastatin exposure. Accordingly, the specific contribution of clopidogrel cannot be isolated from the available data.

The biological mechanism remains unknown. Experimental and review literature suggests that purinergic signaling may contribute to ocular hydrodynamics, including aqueous humor regulation and trabecular meshwork physiology (14, 15). Ciliary blood flow is also linked to aqueous humor production (16). However, there is currently no direct human evidence linking systemic clopidogrel exposure to symptomatic IOP dysregulation. Therefore, the proposed P2Y12–purinergic–aqueous humor pathway should be viewed only as an exploratory hypothesis for future research, not as a mechanistic conclusion supported by the present case.

The clinical implication of this case is limited but potentially relevant. For patients with pre-existing ocular hypertension or angle crowding who develop unexplained ocular discomfort, eye pain, or blurred vision during clopidogrel-containing antiplatelet therapy, ophthalmic referral may be considered for multi-time-point IOP assessment, particularly when clinically evident hemorrhagic causes have not been identified. However, the present case does not justify routine screening or any change in antiplatelet therapy without individualized cardiovascular and ophthalmic assessment.

## 6 Limitations

This report has several important limitations. First, baseline 24-h IOP monitoring before clopidogrel exposure was unavailable; therefore, the marked fluctuation documented during recurrent symptomatic exposure could not be confirmed to represent worsening relative to the patient's baseline diurnal IOP pattern. Second, formal gonioscopy with Shaffer grading and peripheral anterior synechiae assessment was not performed, and angle configuration was not reassessed during the symptomatic phase; therefore, intermittent angle closure or other dynamic angle-related mechanisms could not be excluded. Third, although no clinically evident retinal or optic disc hemorrhage was identified on available ultra-widefield fundus photographs, occult hemorrhagic or subtle structural changes could not be fully excluded because B-scan ultrasonography, symptomatic-phase ultrasound biomicroscopy, and macular OCT were unavailable. Finally, recent patent foramen ovale closure, post-operative physiological stress, natural IOP variability on a background of ocular hypertension and angle crowding, aspirin co-administration, dual antiplatelet therapy, atorvastatin exposure, and concomitant medications remain potential confounders. Accordingly, this case should be interpreted as a dechallenge–rechallenge-supported temporal association and hypothesis-generating pharmacovigilance signal rather than evidence of a definitive clopidogrel-induced effect.

## 7 Conclusion

This case describes recurrent ocular symptoms and objectively documented marked 24-h IOP fluctuation during clopidogrel exposure in a patient with pre-existing ocular hypertension and angle crowding features. Because baseline 24-h IOP monitoring was unavailable, formal gonioscopy was not performed and symptomatic-phase angle reassessment was unavailable, and several confounding factors were present, the findings should be interpreted primarily as a dechallenge–rechallenge-supported temporal association rather than evidence of a definitive clopidogrel-induced effect. This observation may serve as a hypothesis-generating pharmacovigilance signal and suggests that ophthalmic assessment, including multi-time-point or 24-h IOP monitoring when feasible, may be considered in susceptible patients who develop unexplained ocular symptoms during antiplatelet therapy.

## 8 Patient perspective

The patient reported that recurrent ocular symptoms after surgery negatively affected daily comfort and caused concern about the post-operative medication regimen. She noticed improvement after clopidogrel discontinuation and recurrence after resumption, which increased her awareness of a possible medication-related association. She agreed to publication in the hope that it may help clinicians recognize similar cases earlier.

## Data Availability

The original contributions presented in the study are included in the article/Supplementary material, further inquiries can be directed to the corresponding author.
