# Supplementary material for: Symptomatic intraocular pressure dysregulation during clopidogrel exposure after patent foramen ovale closure in a patient with ocular hypertension and angle crowding: a Case Report
Source: Front Med (Lausanne). 2026 Jul 16;13:1836893. doi: 10.3389/fmed.2026.1836893 (PMC13422524; doi:10.3389/fmed.2026.1836893)
Supplement: Supplementary file 1 [file Supplementary_file_1.docx]

**Supplementary Table S1. Itemized Naranjo score used as a structured pharmacovigilance aid**

| Naranjo item | Question | Options (score) | Selection in this case | Score |
| --- | --- | --- | --- | --- |
| 1 | Are there previous conclusive reports on this reaction? | Yes (+1) / No (0) / Unknown (0) | Unknown | 0 |
| 2 | Did the adverse reaction appear after the suspected drug was administered? | Yes (+2) / No (−1) / Unknown (0) | Yes | +2 |
| 3 | Did the adverse reaction improve when the drug was discontinued or a specific antagonist was administered? | Yes (+1) / No (0) / Unknown (0) | Yes | +1 |
| 4 | Did the adverse reaction reappear when the drug was re-administered? | Yes (+2) / No (−1) / Unknown (0) | Yes | +2 |
| 5 | Are there alternative causes that could on their own have caused the reaction? | Yes (−1) / No (+2) / Unknown (0) | Unknown | 0 |
| 6 | Did the reaction reappear when a placebo was given? | Yes (−1) / No (+1) / Unknown (0) | Not performed | 0 |
| 7 | Was the drug detected in blood or other fluids at concentrations known to be toxic? | Yes (+1) / No (0) / Unknown (0) | Not measured | 0 |
| 8 | Was the reaction more severe when the dose was increased, or less severe when the dose was decreased? | Yes (+1) / No (0) / Unknown (0) | Unknown | 0 |
| 9 | Did the patient have a similar reaction to the same or similar drugs in any previous exposure? | Yes (+1) / No (0) / Unknown (0) | Unknown | 0 |
| 10 | Was the adverse event confirmed by any objective evidence? | Yes (+1) / No (0) / Unknown (0) | Yes | +1 |

**Note:** The Naranjo score was used only as a structured pharmacovigilance aid to describe the temporal dechallenge–rechallenge pattern. Alternative causes were not considered excluded because of pre-existing ocular hypertension, angle crowding, recent cardiovascular intervention, postoperative context, and concomitant antiplatelet therapy. Therefore, the score was not interpreted as evidence of definitive causality.

**Supplementary Figure S1. Baseline Humphrey 24-2 visual field testing
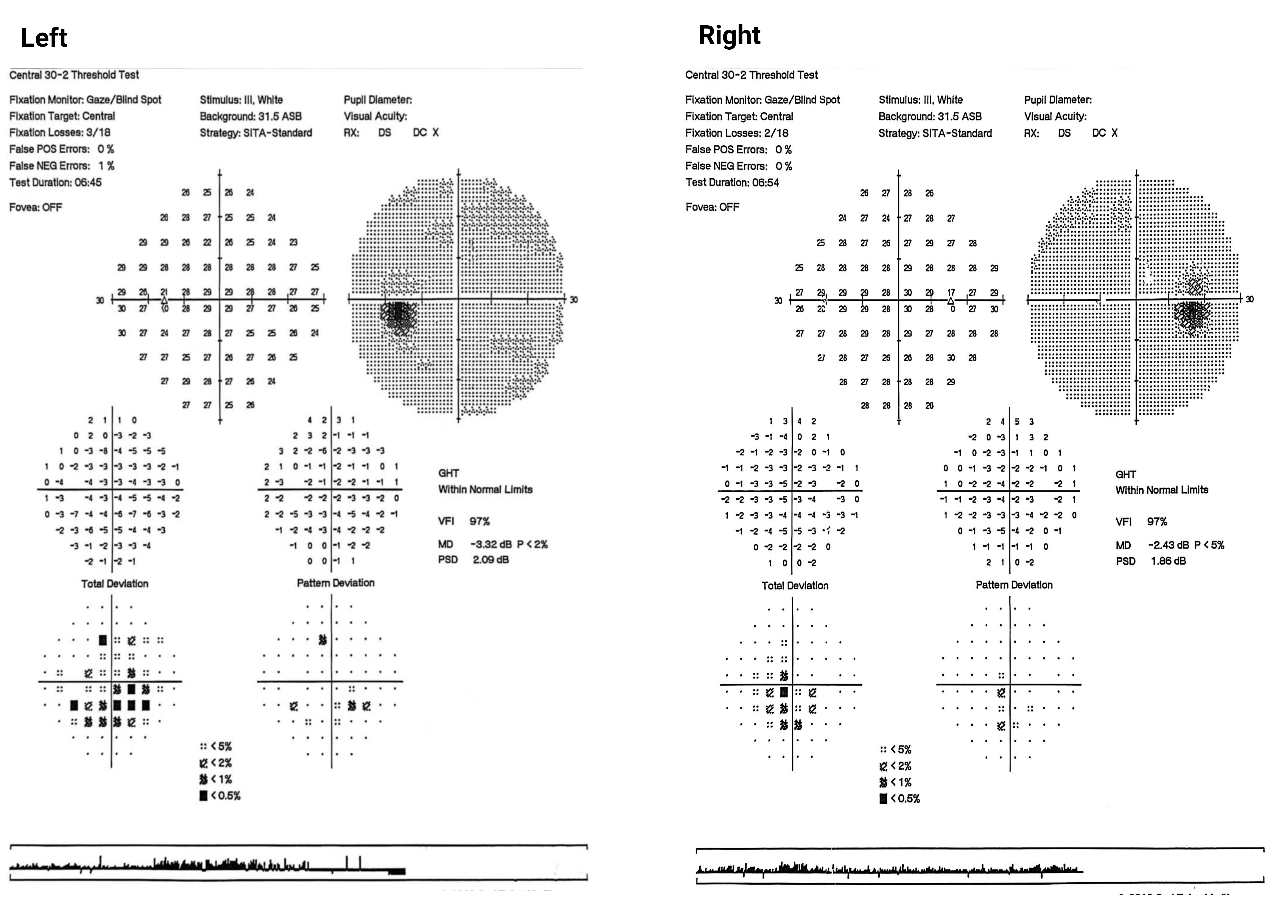
**

**Note**: Humphrey 24-2 visual field testing performed before clopidogrel exposure showed no definite glaucomatous functional defect in either eye.

**Supplementary Figure S2. Baseline Corvis ST assessment.
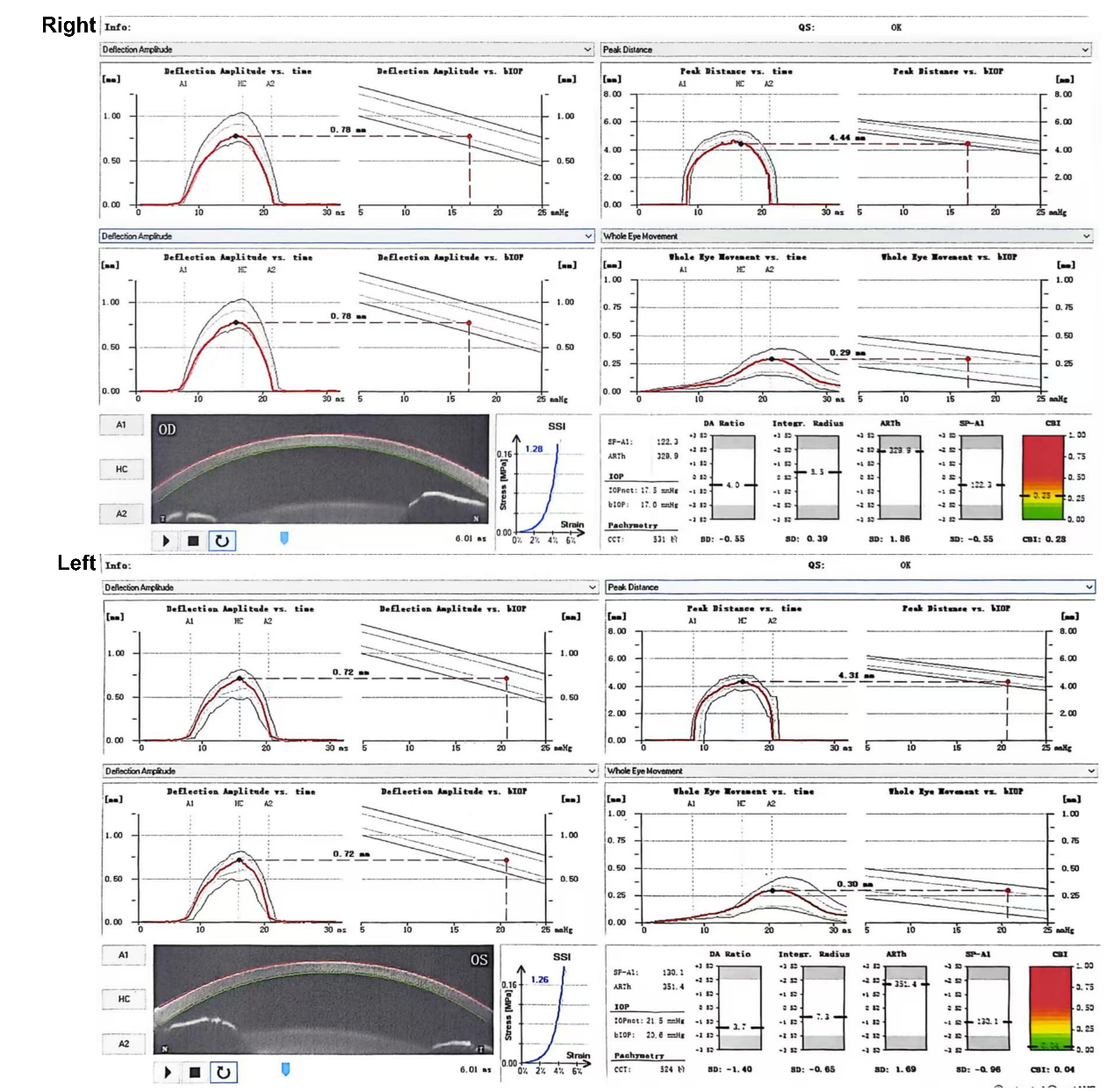
**

**Note:** Corvis ST provided baseline biomechanically corrected IOP and central corneal thickness measurements before clopidogrel exposure.

**Supplementary Figure S3. Baseline ultra-widefield fundus photographs
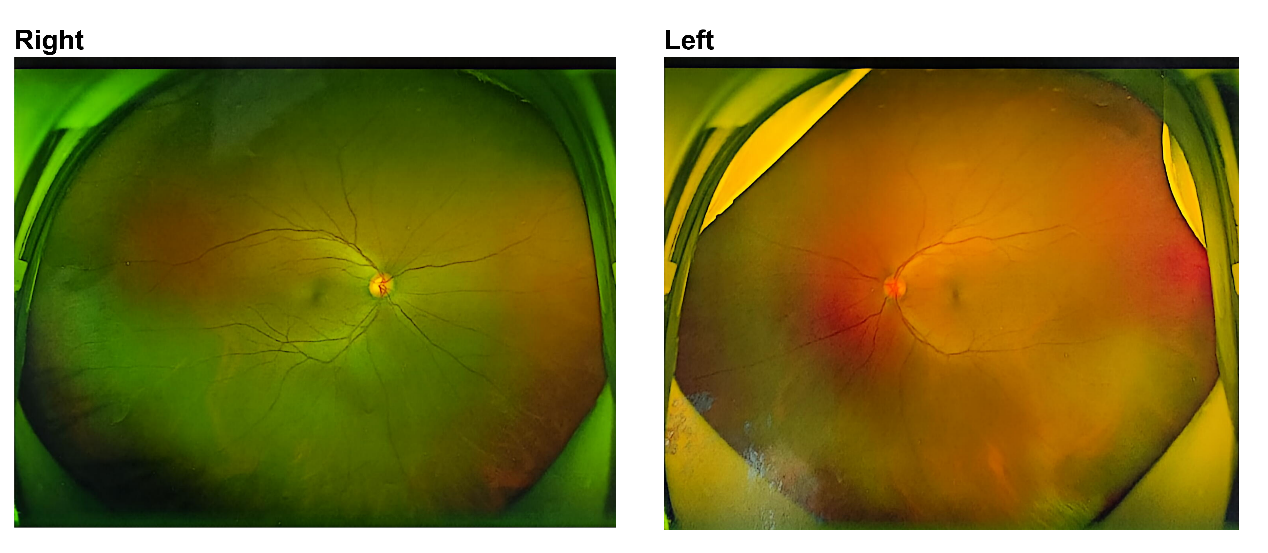
**

**Note:** Available ultra-widefield fundus photographs showed no clinically evident retinal or optic disc hemorrhage.

**Supplementary Figure S4. Detailed 24-hour intraocular pressure measurements**
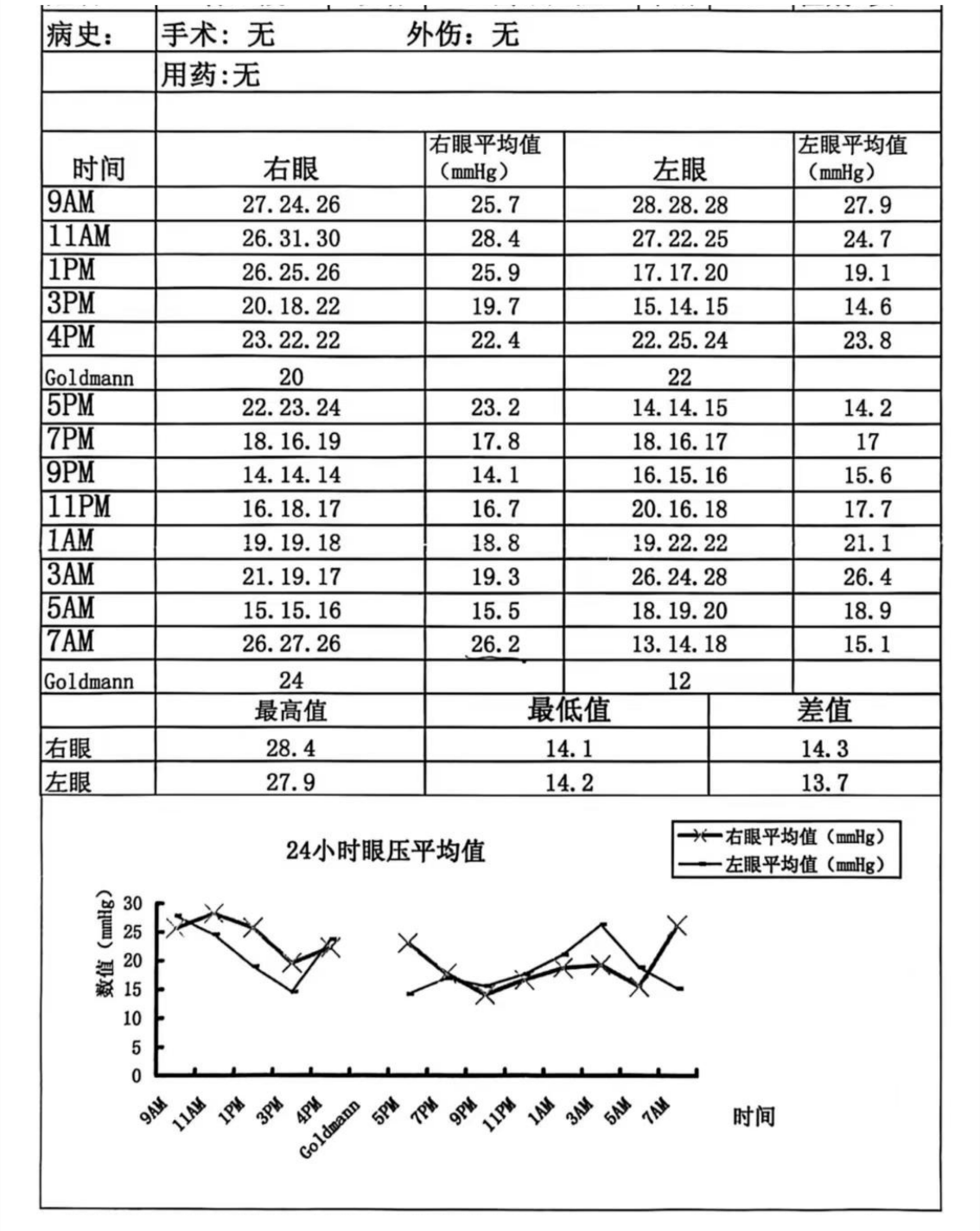


**Note:** Time-point-specific IOP measurements are shown for both eyes during recurrent symptomatic clopidogrel exposure. Baseline 24-hour IOP monitoring before clopidogrel exposure was unavailable; therefore, worsening relative to the patient’s baseline diurnal fluctuation pattern could not be confirmed.

**Supplementary Figure S5. Clinical timeline.**

**
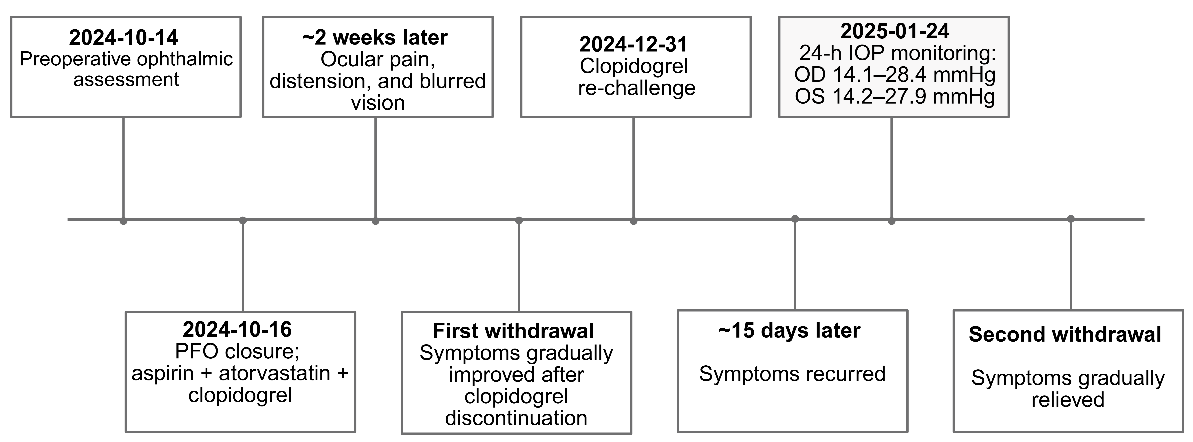
**

**Note:** Timeline showing clopidogrel exposure, symptom onset, withdrawal, re-administration, recurrent symptoms, 24-hour IOP monitoring, and subsequent follow-up.
